# Induction of high affinity monoclonal antibodies against SARS-CoV-2 variant infection using a DNA prime-protein boost strategy

**DOI:** 10.1186/s12929-022-00823-0

**Published:** 2022-06-09

**Authors:** Chen-Yi Chiang, Mei-Yu Chen, Chia-Wei Hsu, Chia-Yeh Liu, Yu-Wen Tsai, Hung-Chun Liao, Jia-Ying Yan, Zih-Shiuan Chuang, Hsin-I. Wang, Chien-Hsiung Pan, Chia-Yi Yu, Guann-Yi Yu, Ching-Len Liao, Shih-Jen Liu, Hsin-Wei Chen

**Affiliations:** 1 National Institute of Infectious Diseases and Vaccinology, National Health Research Institutes, Miaoli, 35053 Taiwan; 2 Department of Life Sciences, National Tsing Hua University, Hsinchu, 30072 Taiwan; 3 Graduate Institute of Biomedical Sciences, China Medical University, Taichung, 406040 Taiwan; 4 Graduate Institute of Medicine, Kaohsiung Medical University, Kaohsiung, 307378 Taiwan

**Keywords:** COVID-19, Monoclonal antibody, Neutralization, SARS-CoV-2, Variant

## Abstract

**Background:**

Calls for the coronavirus to be treated as an endemic illness, such as the flu, are increasing. After achieving high coverage of COVID-19 vaccination, therapeutic drugs have become important for future SARS-CoV-2 variant outbreaks. Although many monoclonal antibodies have been approved for emergency use as treatments for SARS-CoV-2 infection, some monoclonal antibodies are not authorized for variant treatment. Broad-spectrum monoclonal antibodies are unmet medical needs.

**Methods:**

We used a DNA prime-protein boost approach to generate high-quality monoclonal antibodies. A standard ELISA was employed for the primary screen, and spike protein-human angiotensin-converting enzyme 2 blocking assays were used for the secondary screen. The top 5 blocking clones were selected for further characterization, including binding ability, neutralization potency, and epitope mapping. The therapeutic effects of the best monoclonal antibody against SARS-CoV-2 infection were evaluated in a hamster infection model.

**Results:**

Several monoclonal antibodies were selected that neutralize different SARS-CoV-2 variants of concern (VOCs). These VOCs include Alpha, Beta, Gamma, Delta, Kappa and Lambda variants. The high neutralizing antibody titers against the Beta variant would be important to treat Beta-like variants. Among these monoclonal antibodies, mAb-S5 displays the best potency in terms of binding affinity and neutralizing capacity. Importantly, mAb-S5 protects animals from SARS-CoV-2 challenge, including the Wuhan strain, D614G, Alpha and Delta variants, although mAb-S5 exhibits decreased neutralization potency against the Delta variant. Furthermore, the identified neutralizing epitopes of monoclonal antibodies are all located in the receptor-binding domain (RBD) of the spike protein but in different regions.

**Conclusions:**

Our approach generates high-potency monoclonal antibodies against a broad spectrum of VOCs. Multiple monoclonal antibody combinations may be the best strategy to treat future SARS-CoV-2 variant outbreaks.

**Supplementary Information:**

The online version contains supplementary material available at 10.1186/s12929-022-00823-0.

## Background

In the past two decades, outbreaks of two highly pathogenic coronaviruses, SARS-CoV-1[1] and Middle East respiratory syndrome (MERS-CoV)[2], occurred in 2002–2003 and 2012, respectively. Both viruses caused a regional pandemic and led to high morbidity and mortality rates in humans. Severe acute respiratory syndrome coronavirus 2 (SARS-CoV-2) has caused the global coronavirus disease (COVID-19) pandemic since 2019, and it has not been fully controlled for more than 2 years. SARS-CoV-2 continues to mutate, resulting in more than 400 million confirmed infections and approximately 6 million deaths[3]. Currently, widely licensed therapies to prevent or treat COVID-19 are unavailable. Therefore, the development of preventive and therapeutic reagents to combat SARS-CoV-2 infection is a top priority.

SARS-CoV-2 is a Betacoronavirus whose entry into host cells is mediated by a glycosylated spike protein (S) that binds to the angiotensin-converting enzyme 2 (ACE2) receptor[4]. When SARS-CoV-2 attaches to a cell, the spike protein is cleaved into S1 (14–685 residues) and S2 (686–1273 residues) fragments by host proteases. The S1 protein includes the N-terminus (NTD, residues 14–305) and receptor-binding domain (RBD, residues 319–541), while the S2 protein (residues 1237–1273) facilitates membrane fusion and allows the viral genetic material to enter the cell[5]. Substitution of amino acids generates new viral variants. The RBD is a research hotspot. Over the past two years, mutations in the RBD of SARS-CoV-2 variants have been identified in the UK (Alpha, B.1.1.7)[6, 7], South Africa (Beta, B.1.351)[8, 9], Brazil (Gamma, P.1)[10, 11] and India (Delta, B.1.617.2)[12, 13], causing a serious epidemic. Recently, Omicron (B.1.1.529) originated in Botswana, and the epidemic situation in various countries has progressed rapidly[14]. Mutations of S residues that affect ACE2 binding and recognition of antibodies are also associated with enhanced transmission and infectivity[15]. Therefore, RBD is a specific target for the development of many potent neutralizing antibodies and therapeutic agents[16, 17].

In the past 2 years, human monoclonal antibodies from patients infected with SARSCoV-2 have been isolated to neutralize the viruses or treat viral infection[17, 18]. The regions recognized by these antibodies are all located on the RBD of SARS-CoV-2. The high RBD mutation rate of SARS-CoV-2 often affects the recognition of antibodies. In previous studies, a cocktail of antibodies against the SARS-CoV-2 spike protein prevented the virus from rapidly mutating to escape neutralization[19]. Currently, several anti-SARS-CoV-2 mAbs, bamlanivimab (LY-CoV555), etesevimab (LY-CoV016), casirivimab (REGN10933), and imdevimab (REGN10987), have received Emergency Use Authorization (EUA) from the U.S. Food and Drug Administration (FDA) for the treatment of patients with confirmed SARS-CoV-2 infection and mild to moderate COVID-19. The FDA has authorized antibody combinations to achieve beneficial therapies, such as the U.S. FDA emergency authorization of bamlanivimab combined with etesevimab or casirivimab and imdevimab in the treatment of mildly to moderately ill patients over 12 years of age. Authorization of these monoclonal antibody therapies may help patients avoid hospitalization and alleviate the burden on our health care system. However, SARS-CoV-2 continues to mutate, and some antibodies are not authorized to treat new variant infections; thus, the development of new monoclonal antibodies against SARS-CoV-2 is urgently needed.

Vaccines and chemical drugs are produced to fight viral infections. For the same reason, combining vaccination with neutralizing monoclonal antibodies should also prevent outbreaks caused by new variants. Mouse monoclonal antibodies have been developed for many years and diverse antibodies have been quickly obtained and applied to virus infection or treatment-related research in animal models[20–22]. The DNA prime-protein boost immunization approach has been shown to generate high affinity monoclonal antibodies[23, 24]. In the present study, we employed this strategy to generate monoclonal antibodies against the SARS-CoV-2 spike protein. Several monoclonal antibodies were screened by performing ACE2 competition assays and epitope analyses. We utilized monoclonal antibodies to neutralize SARS-CoV-2 variants and used a hamster animal model to understand antibody protection by performing pathological analyses. The epitopes were further determined to confirm that the antibody recognizes the SARS-CoV-2 RBD region. In the future, antibodies that bind to different antigenic epitopes will hopefully be applied to protect infected animals and treat viral infections.

## Methods

### Mouse immunization

BALB/c mice were obtained from the National Laboratory Animal Breeding and Research Center (Taipei, Taiwan). Six- to eight-week-old BALB/c mice were injected intramuscularly with the pSARS-2 DNA vaccine[25] (100 μg per mouse) in hind legs at a 3-week interval, followed by electroporation with a BTX electroporator (ECM830) using two-needle array electrodes (5-mm diameter (BTX 45-0121). Electroporation was performed at 75 V with 10 pulses at 50 ms/pulse and 100-ms intervals between pulses. After two DNA vaccinations, each mouse was immunized twice with 100 μg of SARS-CoV-2 S protein (ACROBiosystems, DE) via subcutaneous injection at a three-week interval. One week after the fourth immunization, blood was collected from the cheek fossa of the mouse and tested using ELISA. Blood samples were collected from the submandibular vein of mice. All animals were housed at the Animal Center of the National Health Research Institutes (NHRI) and maintained in accordance with institutional animal care protocols. All animal experimental protocols were approved by the Institutional Animal Care and Use Committee (IACUC) of the NHRI (Protocol No: NHRI-IACUC-109077-A).

### Cell fusion and hybridoma screening

The mouse that had a higher titer was injected intravenously with 100 μg of SARS-CoV-2 S protein. Three days later, splenocytes were isolated from the immunized mouse and mixed with murine FO myeloma cells at a ratio of 5:1 with ClonaCell^Tm^-HY PEG according to the ClonaCell HY Cloning Kit protocols. The splenocytes were resuspended in fusion recovery medium, incubated at 37 °C for 20 h, and hybridoma selection and cloning were performed simultaneously using methylcellulose-based semisolid medium containing HAT, such as ClonaCell™-HY Medium D. The fused cells were transferred into 10-cm plates and incubated at 37 °C for 10–14 days. After 14 days, the colonies were visible with the naked eye. Each clone was pipetted into an individual well of a 96-well tissue culture plate containing ClonaCell™-HY Medium E and incubated at 37 °C for 3–4 days. The positive hybridoma cells were determined using ELISA. The positive clone with a high titer was chosen for subcloning.

### S proteins of SASR-CoV-2 variants and other human coronaviruses

HEK293 cells expressing S protein of SARS-CoV-1 (SPD-S52H6) or S1 (40591-V08H), RBD_319-537_ (SPD-C52H3), and S proteins (SPN-C52H4) of original SARS-CoV-2, Alpha (SPN-C52H6), Gamma (SPN-C52Hg), Kappa (SPN-C52Hr), and Lambda (SPN-C52Hs) were purchased from ACROBiosystems. The recombinant S proteins of SARS-CoV-2 WA1, Beta, Delta and S_331-524_ (N331-V524) were prepared in the ExpiCHO™ cell expression system. Briefly, the S proteins of SARS-CoV-2 variants were cloned into the clinically used vector pcDNA3.1 and expressed in ExpiCHO™ cells as previously described[26]. The spike gene of SARS-CoV-2 (accession number: MN908947) was synthesized and modified by replacing the residues. The first modification of the spike protein in the cleavage site (residues RRAR _682–685_) between the S1 and S2 domains was replaced with GSAS. Second, the stability of the trimeric spike protein was increased by replacing two prolines at positions 986 and 987[27, 28]. The spike genes from other human coronaviruses, MERS-CoV (accession number: KJ782549.1), human OC43 (accession number: KF572815.1), HKU1 (accession number: DQ437607.1), 229E (accession number: AB691763.1), and NL63 (accession number: KM055633.1), were optimized and synthesized into the pCIneo vector with an HA-tag sequence at the 3’ end of the genes by AllBio Science Inc. The chimeras of SARS-CoV-2 and SARS-CoV-1 were synthesized and subcloned into pcDNA3.1 with a His-tag at the 3’ end of genes, as previously described[29]. All plasmids were transformed into DH5α *E. coli* for plasmid amplification. Plasmids were extracted and purified using an endotoxin-free Qiagen column system (Germany). Proteins were expressed in 293T cells using PolyJet™ reagent (SignaGen Laboratories) according to the manufacturer’s protocol.

### ACE2 competition ELISA

Microplates (96-well) were coated with 8 μg/ml SARS-CoV-2 S proteins at 4 °C overnight. The plates were washed and blocked with blocking buffer at 37 °C for 1 h. After washing, serially diluted anti-SARA-CoV-2 sera, supernatants of hybridoma cells, or purified SARS-CoV-2 mAbs mixed with 5 nM biotinylated human ACE2 (BIOSS, MA) were added to the wells. The plates were incubated at 37 °C for 2 h. After washing, streptavidin-HRP working solution was added to each well for 1 h at 37 °C. Finally, the reactions were developed and the absorbance was measured using a microplate reader at 450 nm.

### SARS-CoV-2 neutralization assay

The virus neutralization assay was conducted in a biosafety level 3 (BSL-3) laboratory and was approved by the Taiwan CDC. The strains of SARS-CoV-2, hCoV-19/Taiwan/4/2020 (WA1), hCoV-19/Taiwan/78/2020 (D614G, B.1.1.515), hCoV-19/Taiwan/729/2020 (Alpha, B.1.1.7), hCoV/Taiwan/1013 (Beta, B.1.351), hCoV-19/Taiwan/906/2020 (Gamma, P.1), and hCoV/Taiwan/1144/2020 (Delta, B.1.617.2) were obtained from the Centers for Disease Control (CDC) in Taiwan. Viruses were amplified in Vero cells and grown in M199 medium supplemented with 2 μg/mL TPCK-trypsin (Sigma) at 37 °C. The virus titer was determined by calculating the 50% tissue culture infectious dose (TCID_50_) using a standard method. Briefly, Vero cells were seeded (2.4 × 10^4^ cells/per well) in 96-well plates and cultured in M199 medium supplemented with 5% FBS at 37 °C for 24 h to form a monolayer. The next day, serial two-fold dilutions of SARS-CoV-2 mAbs were incubated with 200 TCID_50_ of SARS-CoV-2 for 2 h at 37 °C. The antibody-virus complexes were added to Vero cell culture monolayers in 96-well plates. The plates were incubated in a CO_2_ incubator at 37 °C for 4 days, after which the cytopathic effect (CPE) was observed microscopically. The neutralization titer was proportional to the highest dilution of SARS-CoV-2 mAbs that prevented infection of 50% of quadruplicate inoculations.

### Animal experiments

Syrian hamsters (n = 5–6 per group) were challenged intranasally with 1 × 10^5^ TCID_50_ (WA1, D614G, and Alpha), or 1 × 10^3^ TCID_50_ (Delta) SARS-CoV-2 in 50 μl under isoflurane anesthesia. After 3 h, each hamster was injected with 1 mg or 5 mg of mAb-S5[30]. Half-hamsters in each group were sacrificed at Day 3 after challenge to quantify the viral load. Left lung tissues were homogenized in 2 ml of PBS using a gentleMACS® Dissociator (Miltenyi Biotec) to determine the viral load in the lung. After centrifugation at 600 × g for 5 min, the clarified supernatant was harvested for live virus titration (TCID_50_ assay) and viral RNA quantification. The protocols were performed as previously described[31]. RNA was extracted from the tissue supernatant after lysis with TRIzol LS (Ambion), and 10 ng of the RNA was used as a template for RT-qPCR. RT–qPCR was performed on a QuantStudio 6 Flex Real-Time PCR System (ABI) using the KAPA PROBE FAST Universal One-Step qRT–PCR kit (KR1282, Roche). The primers and probes were designed to specifically amplify two target regions: SARS-CoV-2-specific E (E_Sarbeco Forward: ACAGGTACGTTAATAGTTAATAGCGT, E_Sarbeco Reverse: ATATTGCAGCAGTACGCACACA, and E_Sarbeco probe: FAM-ACACTAGCCATCCTTACTGCGCTTCG-BHQ1) and N (CCDC-N forward: GGGGAACTTCTCCTGCTAGAAT, CCDC-N reverse: CAGACATTTTGCTCTCAAGCTG, and CCD-N probe: FAM-TTGCTGCTTGACAGATT-BHQ1) genes. Furthermore, the pathological analysis of lung lobes was performed at Day 6 postchallenge[32]. The 5-μm sections were stained with hematoxylin and eosin for histopathological examinations. Lung tissues were fixed with 4% paraformaldehyde and processed for paraffin embedding. Images were captured using a Leica DFC 5400 digital camera and were processed using Leica Application Suite v.4.13. Pathological severity scores were evaluated according to the percentage of inflammation area for each section from each animal using the following scoring system: 0, no pathological change; 1, affected area ≤ 10%; 2, affected area 10–30%; 3, affected area 30–50%; and 4, affected area ≥ 50%.

### Kinetic analysis

The interaction between S proteins of SARS-CoV-2 variants and mAbs was measured using biolayer interferometry (BLI) on Octet RED (ForteBio, CA). All the interaction analyses were conducted at 25 °C in PBS with 0.05% Tween 20. The anti-mouse IgG Fc capture (AMC) biosensors were preimmobilized with 500 nM purified monoclonal antibodies for 100 s. The 96-well microplates used in the Octet RED were filled with 200 μl of sample or buffer per well and agitated at 1000 × rpm. The loaded biosensors were washed with buffer for 30 s and transferred to wells containing S proteins of SARS-CoV-2 variants at concentrations of 200, 100, 50, 25, 12.5, 6.25, and 3.13 nM in buffer. The association was observed for 400 s, and dissociation of each protein of interest was observed for 500 s in the sample diluent. Kinetic parameters (*K*_on_ and *K*_off_) and affinities (*K*_D_) were calculated with a 1:1 binding model using ForteBio Data Analysis 8.2 software to evaluate the data. Independent measurements were performed 3 times.

### Epitope binning

Epitope binning was performed using an Octet RED instrument. SARS-CoV-2 mAbs in kinetic assay buffer (PBS containing 0.05% Tween 20) were immobilized onto AMC biosensors and saturated with the S proteins. Time 0 represents binding to the mAbs. Complete epitope binning of the five selected SARS-CoV-2 mAbs was evaluated by determining the ability of each pair of antibodies to simultaneously bind S protein using BLI. The complexes were then incubated for 300 s with each of the indicated antibodies. The matrix presents the identified epitope specificity based on the various competition experiments. All data were analyzed using ForteBio Data Analysis 8.2 software.

### Flow cytometry

The FITC-labeled anti-HA antibody and FITC-labeled anti-His antibody were purchased from Biolegend (San Diego, CA). The anti-SARS-CoV-1 S2 antibody (Mab5)[33] and SARS-CoV-2 mAbs were labeled with Alexa Fluor 488 and 647, respectively. Alexa Fluor 488 and 647 Lightning-Link conjugation kits were purchased from Abcam (Cambridge, UK). 293T cells (5 × 10^5^/well) were transfected with 1 μg of the indicated DNA plasmids using PolyJet™ reagent (SignaGen Laboratories) according to the manufacturer’s protocol. At 48 h after transfection, cells were harvested and stained with fluorophore-labeled anti-tag antibodies and mAbs. The spike proteins of human coronaviruses and chimeras of SARS-CoV-1 and SARS-CoV-2 were analyzed using an Attune NxT flow cytometer (Thermo Fisher, MA) and FlowJo V10 software.

### Statistical analysis

Statistical data were generated using GraphPad Prism 8.0.2 software. The statistical significance of differences in results between experimental groups was determined using the Kruskal–Wallis test with Dunn’s multiple comparisons test. Differences were considered statistically significant if the p value was ≤ 0.05.

## Results

### Generation and functional screen of monoclonal antibodies against the spike protein of SARS-CoV-2

BALB/c mice were immunized with the DNA vaccine twice and then boosted with recombinant SARS-CoV-2 spike protein (S) twice. Murine hybridoma technology was used to generate hybridoma cell lines that produced S-specific monoclonal antibodies. After two rounds of standard ELISA screening, 42 clones were selected for S-hACE2 blocking assays. The top 5 blocking clones were selected for further characterization (Additional file 1: Fig. S1A). Four monoclonal antibodies belonged to the IgG2a subclass, while one monoclonal antibody belonged to the IgG2b subclass. All 5 monoclonal antibodies contained a kappa light chain (Additional file 1: Fig S1B).

### Neutralization potency of monoclonal antibodies against multiple SARS-CoV-2 variants

**Fig. 1:**
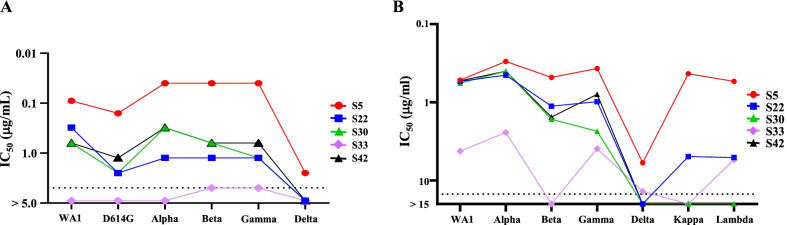
The neutralization and ACE2 competition potency of 5 mAbs against SARS-CoV-2 and its variants. **A** The viruses were mixed with mAbs (a twofold serial dilution), incubated for 2 h, and subsequently cocultured with Vero cells for 4 days. Quadruplicates of each dilution were performed. Cytopathogenic effects of viral infection were visually scored for each well. The results were transformed into the percentage of neutralization at a particular monoclonal antibody concentration. The half-maximal inhibitory concentration (IC_50_) was plotted as a four-parameter dose–response curve using GraphPad Prism 8.0.2 software. The dotted line represents the concentration of 5 μg/mL. **B** Comparisons of the biotinylated human ACE2 protein and mAbs binding to S proteins of SARS-CoV-2 variants measured using ELISA. The IC_50_ values of mAbs against S proteins of SARS-CoV-2 variants are presented. The dotted line represents the concentration of 15 μg/mL

Microneutralization assays were performed to evaluate the neutralizing capacity of 5 monoclonal antibodies against multiple SARS-CoV-2 variants. We first determined the neutralization potency of 5 monoclonal antibodies against SARS-CoV-2 and WA1 (Wuhan-1 strain). Representative wells of microneutralization assays are shown in Additional file 2: Fig. S2A. Except for mAb-S33, the other four mAbs (mAb-S5, mAb-S22, mAb-S30, and mAb-S42) exhibited neutralizing capacity against WA1. We further determined the neutralization potency of 5 mAbs against SARS-CoV-2 variants, including D614G, Alpha, Beta, Gamma, and Delta (Additional file 2: Fig. S2B). The half-maximal inhibitory concentration (IC_50_) of each mAb against SARS-CoV-2 and its variants is summarized in Fig. 1A. mAb-S5 was the most potent clone to block virus infection among the 5 monoclonal antibodies. The IC_50_ values of mAb-S5 for WA-1, D614G, Alpha, Beta, Gamma, and Delta were 0.09 µg/mL, 0.16 µg/mL, 0.04 µg/mL, 0.04 µg/mL, 0.04 µg/mL, and 2.5 µg/mL, respectively. The high potency against the Beta variant is important to treat Beta-like variants in the future.

### Monoclonal antibodies block the binding of spike protein of SARS-CoV-2 variants to human ACE2

**Fig. 2:**
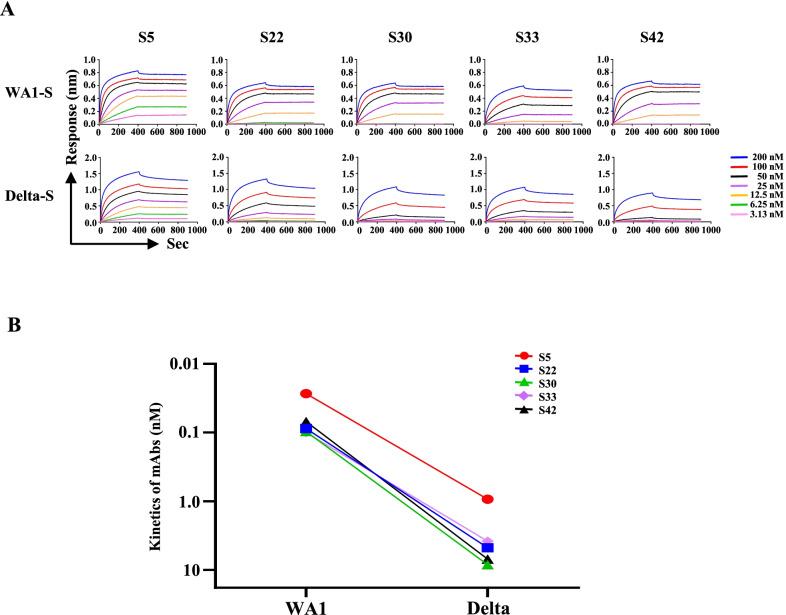
The kinetics of mAbs binding to SARS-CoV-2 variants. **A** The binding profiles of mAbs to trimeric S proteins of SARS-CoV-2 (WA1-S and Delta-S) were analyzed by detecting BLI using an OctetRED instrument. The mAbs were captured by anti-mouse IgG Fc (AMC) sensors immobilized on a chip and tested for binding to gradient concentrations of the S proteins of SARS-CoV-2 variants. The concentrations of SAS-CoV-2 trimeric S proteins ranged from 200 nM to 3.13 nM. Binding kinetics were evaluated using ForteBio Data Analysis 8.2 software. **B** Summary KD of mAbs is shown

Various mutant variants have rapidly spread across a large area of the world. Not all variants were available in our laboratory collection during the preparation of this manuscript. A competition assay was performed to examine the ability of monoclonal antibodies to block the binding of the spike protein of SARS-CoV-2 variants to human ACE2. The data showed that mAb-S5 blocked the binding of all spike proteins of SARS-CoV-2 variants to human ACE2 (Additional file 3: Fig. S3). The IC_50_ values of five mAbs against the spike protein of SARS-CoV-2 Wuhan-1 (WA1-S), Alpha variant (Alpha-S), Beta variant (Beta-S), Gamma variant (Gamma-S), Delta variant (Delta-S), Kappa variant (Kappa-S), and Lambda variant (Lambda-S) are summarized in Fig. 1B. Although the IC_50_ of mAb-S5 against Delta-S (5.45 µg/mL) was higher than WA1-S (0.52 µg/mL), the blocking ability may still be able to protect against Delta variant infection. Since the Delta variant was different from the other variants in the authentic virus neutralizing and spike protein blocking assays, we then determined the binding ability of mAbs to WT-S and Delta-S using biolayer interferometry (Fig. 2A). The equilibrium dissociation constants (KD) of five mAbs against WA1-S and Delta-S are summarized in Fig. 2B. Consistent with the trend of the blocking assay, mAb-S5 showed the strongest binding affinity for WA1-S (KD = 2.7 ± 0.26 × 10^–11^ M) among the five mAbs. Again, although mAb-S5 exhibited reduced binding affinity for Delta-S (KD = 9.3 ± 0.11 × 10^–10^ M) compared with WA1-S, mAb-S5 still displayed high binding affinity for Delta-S. Overall, these results suggest that mAb-S5 recognizes and neutralizes all SARS-CoV-2 and its variants.

### Therapeutic efficacy of mAb-S5 against SARS-CoV-2 variants

**Fig. 3:**
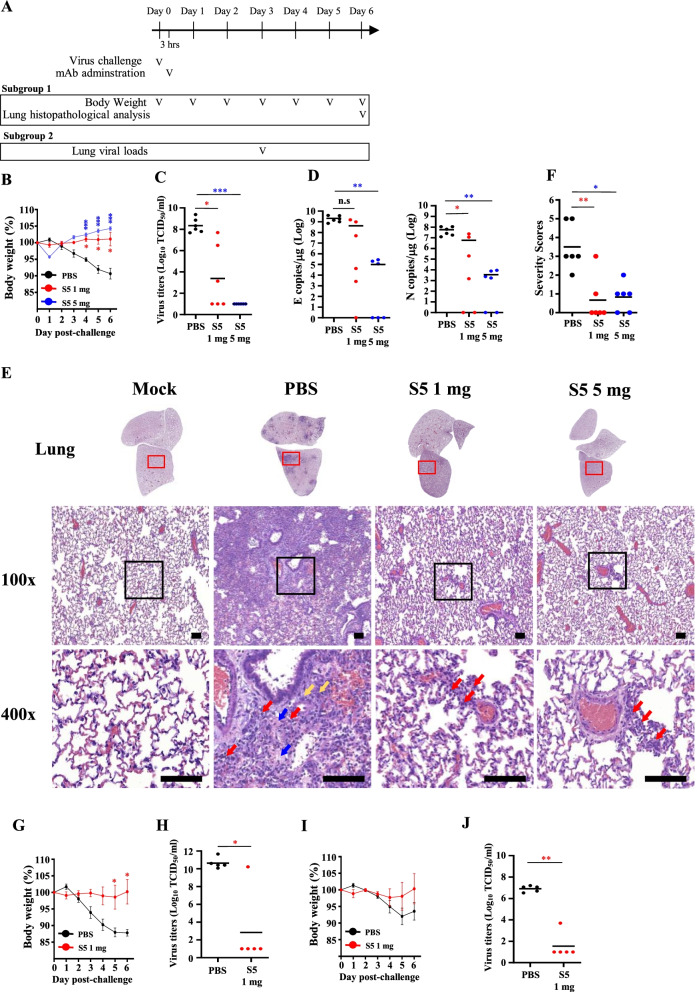
Therapeutic efficacy of mAb-S5 against SARS-CoV-2 (WA1, D614, and Alpha) infection in hamsters. **A** The experimental flow chart is shown. Groups of Syrian hamsters (n = 5–6, 11–12 weeks of age) were intranasally challenged with SARS-CoV-2 (WA1, 1 × 10^5^ TCID_50_ per hamster). After 3 h of challenge, Syrian hamsters were divided into two groups and intraperitoneally administered 1 mg or 5 mg of mAb-S5. Body weights were recorded daily. Body weight changes (%) relative to the day of viral challenge are plotted (**B**). Infectious virus titers **C** and viral RNA copies **D** in the lung were evaluated at Day 3 after virus challenge. **E** Pathological analysis of lung lobes at Day 6 postchallenge using standard HE staining. Red arrows indicate immune cell infiltration. Blue arrows indicate extensive fibrin filling the alveolar space. Yellow arrows indicate pneumocyte hyperplasia. Scale bars represent 100 μm **F**) Pathological severity scores were evaluated according to the percentage of inflammatory area for each section from each animal using the following scoring system: 0, no pathological change; 1, affected area ≤ 10%; 2, affected area 10–30%; 3, affected area 30–50%; and 4, affected area ≥ 50%. Body weight changes (%) relative to the day of D614G **G** and Alpha variant **I** challenge are plotted. Infectious virus titers in the lung measured 3 days after D614G **H** and Alpha **J** challenge are shown. Statistical significance was determined using the Kruskal–Wallis test with Dunn’s multiple comparison test: *p < 0.05, **p < 0.01, and ***p < 0.001

Considering the superior potency of mAb-S5, we further evaluated the therapeutic effects of mAb-S5 on SARS-CoV-2 infection in vivo. Groups of hamsters were treated with PBS or 1 mg or 5 mg of mAb-S5 at 3 h after infection with SARS-CoV-2 (WA1) via the intranasal route. All hamsters were randomly divided into 2 subgroups. One subgroup of hamsters was sacrificed at Day 3 postinfection to evaluate the viral loads in the lung. The body weight of the other subgroup of hamsters was monitored over a 6-day period and then the lung histopathology was examined. The experimental scheme is shown in Fig. 3A. Compared to the PBS-treated group, all hamsters treated with 1 mg or 5 mg of mAb-S5 showed a significantly reduced degree of SARS-CoV-2-induced body weight loss (Fig. 3B). In addition, viral loads in the lungs of mAb-S5-treated hamsters were reduced in a dose-dependent manner, as evaluated by measuring infectious virus titers (Fig. 3C) and viral RNA (Fig. 3D). Moreover, hamsters treated with either 1 or 5 mg of mAb-S5 displayed mild lung damage with only small amounts of immune cell infiltration (red arrow) around the bronchioles and blood vessels at Day 6 after challenge, while the PBS-treated hamsters displayed severely diffuse alveolar damage characterized by thickening of alveolar septa, immune cell infiltration, extensive fibrin filling the alveolar space (blue arrow), and pneumocyte hyperplasia (yellow arrow) (Fig. 3E). Pathological severity scores are summarized in Fig. 3F. Furthermore, we evaluated the therapeutic effects of mAb-S5 on the D614G and Alpha variants. Hamsters treated with 1 mg of mAb-S5 showed reduced body weight loss after D614G or Alpha variant infection (Fig. 3G and I). Consistent with the body weight findings, the infectious virus titers in the lungs of mAb-S5-treated hamsters were substantially reduced (Fig. 3H and J). Based on these results, mAb-S5 may be used for the treatment of diseases caused by SARS-CoV-2 infection.

**Fig. 4:**
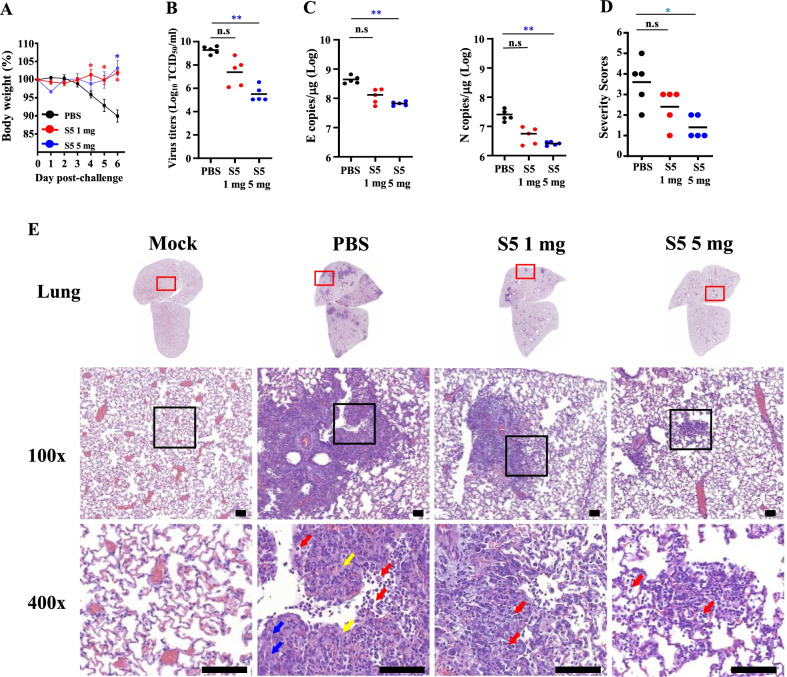
Therapeutic efficacy of mAb-S5 against SARS-CoV-2 (Delta) infection in hamsters. Groups of Syrian hamsters (*n* = 5, 11–12 weeks of age) were intranasally challenged with SARS-CoV-2 (Delta, 1 × 10^3^ TCID_50_ per hamster). After 3 h of challenge, Syrian hamsters were divided into two groups and intraperitoneally administered 1 mg or 5 mg of mAb-S5. Body weights were recorded daily. Body weight changes (%) relative to the day of viral challenge are plotted (**A**). Infectious virus titers **B** and viral RNA copies **C** in the lung were evaluated at Day 3 after virus challenge. **E** Pathological analysis of lung lobes at Day 6 postchallenge measured using standard HE staining. Red arrows indicate immune cell infiltration. Blue arrows indicate extensive fibrin filling the alveolar space. Yellow arrows indicate pneumocyte hyperplasia. Scale bars represent 100 μm. **D** Pathological severity scores were evaluated according to the percentage of inflammatory area for each section from each animal using the following scoring system: 0, no pathological change; 1, affected area ≤ 10%; 2, affected area 10–30%; 3, affected area 30–50%; and 4, affected area ≥ 50%. Statistical significance was determined using the Kruskal–Wallis test with Dunn’s multiple comparison test: *p < 0.05 and **p < 0.01

Among the variants we examined, the neutralizing capacity of mAb5 against the Delta variant was less efficient in the in vitro microneutralization assay. Next, we evaluated the therapeutic potential of mAb-S5 against Delta variants in vivo. The experimental scheme is the same as that described in Fig. 3A. Compared to the PBS-treated group, treatment of hamsters with 1 mg or 5 mg of mAb-S5 resulted in a significantly reduced degree of SARS-CoV-2-induced body weight loss (Fig. 4A). Similar to WA1 challenge studies, viral loads in the lung and severity of lung damage in mAb-S5-treated hamsters were reduced in a dose-dependent manner after Delta challenge. Treatment of hamsters with 5 mg of mAb-S5 significantly reduced infectious virus titers (Fig. 4B), viral RNA levels (Fig. 4C), and lung damage severity (Fig. 4D, E). Thus, mAb-S5 exerts potential therapeutic effects on SARS-CoV-2 variant infection.

### Binding properties and binding site analysis of monoclonal antibodies to the spike protein of SARS-CoV-2

**Fig. 5:**
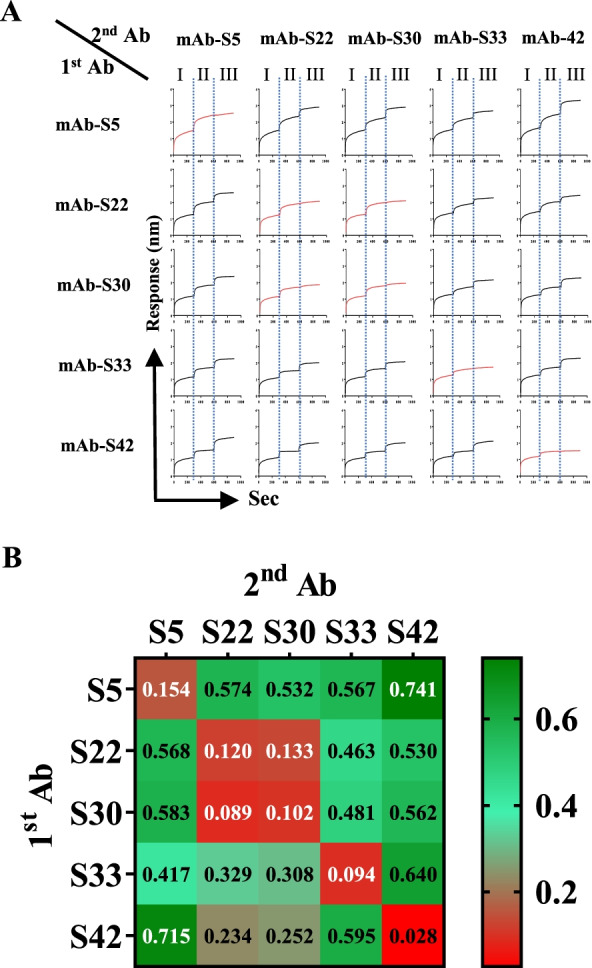
Epitope binning of mAbs for binding to the SARS-CoV-2 S protein. **A** The epitope binning of mAbs was determined by performing bilayer interferometry (BLI). The purified mAbs were immobilized on an AMC sensor and saturated with S protein. Region I represents the mAbs immobilized on sensors, Region II represents the S protein saturated on mAbs, and Region III represents the S protein incubated with each of the indicated antibodies.Each step was incubated for 300 s. **B** Complete epitope binning of the mAbs was evaluated by the ability of each pair of antibodies to simultaneously bind S protein by BLI. The matrix presents the concluded epitope specificity based on the various competition experiments

We used biolayer interferometry technology with a tandem binding model of antibody pairs to bin the epitopes recognized by the five antibodies. As shown in Fig. 5A, regions I, II, and III represent immobilization of the first mAb on sensors, saturation of S protein on the first mAbs, and incubation with the second mAb, respectively. An increase in the signal shift after incubation with the second mAb represents the binding of the second mAb to the S protein without interference by the first bound mAb. Figure 5B summarizes the results for epitope binning of the five mAbs. mAb-S22 and -S30 competed with each other, as marked in red, while mAb-S5, -S33, and -S42 did not compete with the other four mAbs, as marked in green. These results suggest that mAb-S22 and -S30 bind to the S protein at the same (or adjacent) epitope(s). Notably, mAb-S5, -S33, and -S42 bind to the S protein at different epitopes.

**Fig. 6:**
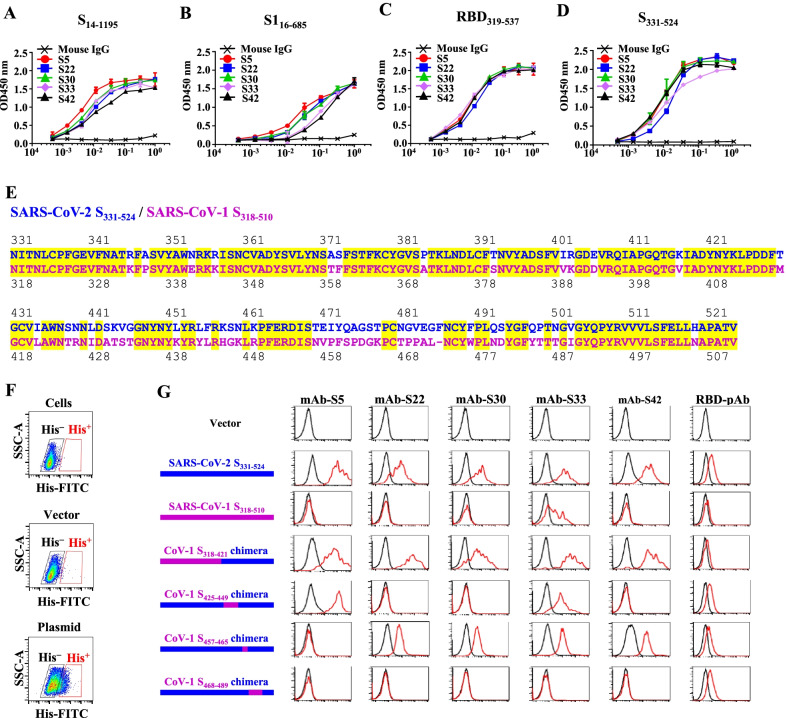
Characterizations of mAbs. S_14-1195_ (**A**), S1_1-667_ (**B**), RBD_319-537_ (**C**), or S_331-524_ **D** were coated on 96-well plates (0.4 μg per well). Three-fold serial dilutions of mAb-S5(●), -S22 (■), -S30 (▲), -S33 (◆), -S42(▲), and control mouse IgG (x) were prepared for standard ELISA. The binding activity of mAbs to S and S fragments was evaluated using an ELISA reader. The results are presented as the means ± standard errors of the means from triplicate wells. **E** Alignment of SARS-CoV-2 S_331-524_ and SARS-CoV-1 S_318-510_. Identical amino acid residues are marked in yellow. Each chimeric expression vector was transfected into 293T cells for 48 h. The cells were harvested and stained with FITC-labeled anti-His and Alexa 647-labeled mAbs. **F** Representative gating strategy for transfected 293T cells. **G** Recognition of each chimeric protein by mAbs was determined using flow cytometry. His^−^ cells: black line; His^+^ cells: red line

Different fragments of recombinant S protein were used as coating antigens for ELISA to analyze the binding regions of the five mAbs. All five mAbs bound well to baculovirus-insect cell-expressed S protein (S_14-1195_) (Fig. 6A), HEK293 cell-expressed recombinant S1_16-685_ (Fig. 6B), receptor binding domain of S (S _319–537_) (Fig. 6C), and CHO cell-expressed recombinant S fragment (S_331-524_) (Fig. 6D). Based on these results, the epitopes of the 5 mAbs are located in the S_331-524_ region. We further examined the specificity of the 5 mAbs. When using SARS-CoV-1 S_306-527_ (equivalent to receptor binding domain of SARS-CoV-1 spike protein) as a coating antigen for ELISA, only mAb-S33 bound to SARS-CoV-1 S_306-527_ (Additional file 4: Fig. S4). None of the 5 mAbs recognized the spike protein of the other human coronaviruses OC43, HKU1, NL63, 229E, and MERS (Additional file 5: Fig. S5). Therefore, mAb-S5, -S22, -S30, and -S42 specifically recognize S_331-524_ of SARS-CoV-2.

Except for mAb-S33, the other 4 mAbs could not bind to SARS-CoV-1 S_306-527_. We hypothesized that mAbs were lost on binding ability when critical recognition regions in S_331-524_ were replaced as counter parts of SARS-CoV-1. Therefore, we constructed plasmids to express S_331-524_ chimeras with various SARS-CoV-1 fragments to further refine the binding regions of the 5 mAbs. Chimeras are indicated on the left side of Fig. 6G. Each recombinant fragment contains a His tag at the C-terminus. Alignment of the amino acid sequences of SARS-CoV-2 S_331-524_ and SARS-CoV-1 S_318-510_ (the counterpart of SARS-CoV-2 S_331-524_) is shown in Fig. 6E. Each expression vector was transfected into 293T cells. His tag^+^ and His tag^−^ transfected cells were gated to analyze reactivity to each mAb (Fig. 6F). Consistent with the results presented in Fig. 6D and S4, all 5 mAbs recognized SARS-CoV-2 S_331-524_-transfected cells. Only mAb-S33, but not the other 4 mAbs, recognized SARS-CoV-1 S_318-510_-transfected cells (Fig. 6G). mAb-S5 recognized the CoV-1 S_318-421_ chimera and CoV-1 S_425-449_ chimera but did not react with the CoV-1 S_457-465_ chimera and CoV-1 S_468-489_ chimera. These results suggest that the epitope of mAb-S5 contains the SARS-CoV-2 S_470-503_ region. Notably, mAb-S22, -S30, and -S42 recognized the CoV-1 S_318-421_ chimera and CoV-1 S_457-466_ chimera, but did not react with the CoV-1 S_425-449_ chimera and CoV-1 S_468-489_ chimera. According to these results, SARS-CoV-2 S_438-462_ and SARS-CoV-2 S_481-503_ are critical recognition regions for mAB-S22, -S30, and -S42. mAb-S33 recognized all the recombinant fragments except for the CoV-1 S_468-489_ chimera. These results suggest that the epitope of mAb-S33 contains the SARS-CoV-2 S_481-503_ region.

## Discussion

In this study, five monoclonal antibodies against SARS-CoV-2 spike protein from generated hybridomas are carefully chosen for further characterization. These antibodies are highly specific for the recognition of SARS-CoV-2 S_331-524_, except that mAb-S33 can recognize both SARS-CoV-1 and SARS-CoV-2 (Fig. 6D, G, and Additional file 4: Fig. S4). In the binning study, mAb-S22 and mAb-S30 interfere reaction with S protein (Fig. 5). Interestingly, mAb-S22 and mAb-S30 show different binding behaviors to Kappa-S and Lambda-S (Fig. 1B). Thus, mAb-S22 and mAb-S30 are different clones and may bind to the S protein at adjacent epitopes. Importantly, mAb-S5, -S33, and -S42 do not interfere with the other 4 mAbs in binding to the S protein (Fig. 5). Therefore, all 5 mAbs recognize different epitopes of the S protein.

Viruses are constantly changing to adapt to the environment. A great concern is that the global pandemic produces new SARS-CoV-2 variants. Emerging variants may be antigenically distinct from the prototype strain, thus resulting in the ineffectiveness of current antibody and vaccine strategies[34]. We report the discovery of mAb-S5 using a DNA prime-protein boost strategy, which is a highly potent neutralizing antibody against authentic variants WA1, D614G, Alpha, and Gamma that blocks ACE2 binding through an interaction with the RBD. These in vitro results indicate that mAb-S5 may have therapeutic effects on COVID-19. After intranasal infection of SARS-CoV-2 with hamsters, SARS-CoV-2 replicated efficiently in the lungs, caused body weight loss, and resulted severe pathological lung lesions. These findings show that hamster infection model is a useful animal model for testing vaccines and antiviral treatments[32, 35]. To proof-of-concept, hamsters were treated with mAb-S5 after SARS-CoV-2 challenge. We showed excellent in vivo therapeutic protection of mAb-S5 against WA1, D614G, and Alpha variants in the hamster challenge model, as indicated as restoration of body weight, reduction of viral load in the lungs, and lung pathology manifestation (Fig. 3). Although mAb-S5 is less efficient at neutralizing the Delta variant in vitro (Fig. 1A), it still exerts therapeutic effects on protecting hamsters from diseases caused by Delta variant infection (Fig. 4). Therefore, mAb-S5 retains full potency against the newly emerging variants we examined.

In this study, antibodies are treated at 3 h post-infection which only provide a solid proof-of-concept results. However, treatment with antibody after symptom development may be more clinically relevant. For example, hamsters administrate antibodies at 1- or 2-day post-infection. As research on emerging viral infections is still developing, the experimental procedures should be revised in the future study to close to clinically relevant.

The recognition region of mAb-S5 is located in S_331-524_ (Fig. 6D) and has been further refined within S_470-503_ (Fig. 6G). Alignment of amino acid sequences within S_470-503_ of variants with the original Wuhan reference virus reveals several mutations: N501Y in the Alpha variant, E484K/N501Y in the Beta and Gamma variants, E484Q in the Kappa variant, and F490S in the Lambda variant. The authentic virus neutralizing capacity (Fig. 1A), blockade of spike protein binding to ACE2 (Fig. 1B), binding to spike protein (Fig. 2), and therapeutic effect (Fig. 3) of mAb-5 seem unaffected by these mutations. However, only one mutation, T478K, within S_470-503_ was identified in the Delta variant. This mutation leads to substantial effects on mAb-S5 recognition of the spike protein. These results suggest that T478K is critical for mAb-S5 recognition.

During the manuscript preparation, Omicron variant became prevalent in the world. We examined the neutralization potency of 5 mAbs against Omicron-pseudovirus. The mAb-S22 showed the better ability than the other 4 mAbs to block Omicron-pseudovirus infection (Additional file 6: Fig. S6). Consistent with this finding, mAb-S22 is more potent than the other 4 mAbs to block the binding of the spike protein of Omicron variant to human ACE2 (Additional file 7: Fig. S7). These results suggest that mAb22 may have protective effects against the Omicron variant.

Several potent neutralizing antibodies are in clinical use or in clinical trials. The following 8 mAbs have been approved for emergency use authorization to treat patients with COVID-19 presenting a high risk of severe illness: bamlanivimab (LY-CoV555), etesevimab (LY-CoV016), casirivimab (REGN10933), imdevimab (REGN10987), cilgavimab (COV2-2130), tixagevimab (COV2-2196), sotrovimab (VIR-7831), and regdanvimab (CT-P59)[36]. However, emerging variants exhibit increased resistance to these antibodies. Bamlanivimab (LY-CoV555), etesevimab (LY-CoV016), and casirivimab (REGN10933) are reported to be sensitive to the mutations E484K and L452R, K417N/T, and E484K and K417N, respectively[37–39]. Furthermore, the B.1.351 variant, which carries K417N/E484K/N501Y mutations in S_331-524_, is refractory to neutralization by bamlanivimab (LY-CoV555), etesevimab (LY-CoV016), and casirivimab (REGN10933)[37]. We show that mAb-S5 is potent in blocking Beta-S binding to ACE2, in which the IC_50_ of the Beta variant (0.47 μg/mL) is lower than the IC_50_ of WA1 (0.52 μg/mL). Importantly, mAb-S5 effectively neutralized the Beta variant (TW/1013, B.1.351). The IC_50_ of the Beta variant (TW/1013) was reduced twofold compared to the IC_50_ of WA1 (TW/4). These results suggest that K417N/E484K/N501Y mutations do not affect the potency of mAb-S5. Imdevimab (REGN10987) is sensitive to K444Q and V445A mutations[19]. The CoV-1 S_425-449_ chimera substitutes S_438-462_ with SARS-CoV-1, which is recognized by mAb-S5 (Fig. 6G). Based on these results, S_438-462_ is not a critical region for mAb-S5 recognition. Thus, mAb-S5 is different from imdevimab (REGN10987) and is not sensitive to the K444Q and V445A mutations. The combination of different antibodies may prevent rapid neutralization-escaping mutants. Therefore, mAb-S5 is a potential candidate for inclusion in the antibody cocktail for COVID-19 treatment.

## Conclusions

Our approach generates high-potency monoclonal antibodies against a broad spectrum of VOCs. Multiple monoclonal antibody combinations may be the best strategy to treat future SARS-CoV-2 variant outbreaks.

## Supplementary Information

**Additional file 1: Figure S1.** Selection and characterization of monoclonal antibodies against SARS-CoV-2 spike protein.

**Additional file 2: Figure S2.** The neutralization potency of 5 mAbs against SARS-CoV-2 and its variants.

**Additional file 3: Figure S3.** Competition of human ACE2 protein with mAbs targeting the S proteins of SARS-CoV-2 variants.

**Additional file 4: Figure S4. **Characterization of mAbs against recombinant SARS-CoV-1 RBD306-527.

**Additional file 5: Figure S5.** Characterization of mAbs against human other coronaviruses.

**Additional file 6: Figure S6.** The neutralization potency of 5 mAbs against Omicron pseudovirus.

**Additional file 7: Figure S7.** Competition of human ACE2 protein with mAbs targeting the S protein of Omicron variant.

## Data Availability

The datasets analyzed in the current study are available upon reasonable request.

## Abbreviations

COVID-19: Coronavirus disease
SARS-CoV-1: Severe acute respiratory syndrome coronavirus 1
MERS-CoV: Middle East respiratory syndrome
SARS-CoV-2: Severe acute respiratory syndrome coronavirus 2
WA1: Wuhan strain
RBD: Receptor binding domain
NTD: N-terminal domain
ACE2: Angiotensin-converting enzyme 2
VOCs: Variants of concern
FDA: Food and Drug Administration
EUA: Emergency Use Authorization
