# Supplementary material for: Induction of high affinity monoclonal antibodies against SARS-CoV-2 variant infection using a DNA prime-protein boost strategy
Source: J Biomed Sci. 2022 Jun 9;29:37. doi: 10.1186/s12929-022-00823-0 (PMC9178533; doi:10.1186/s12929-022-00823-0)
Supplement: Supplementary file 1 — Additional file 1: Figure S1. Selection and characterization of monoclonal antibodies against SARS-CoV-2 spike protein. [file 12929_2022_823_MOESM1_ESM.pdf]

Figure S1

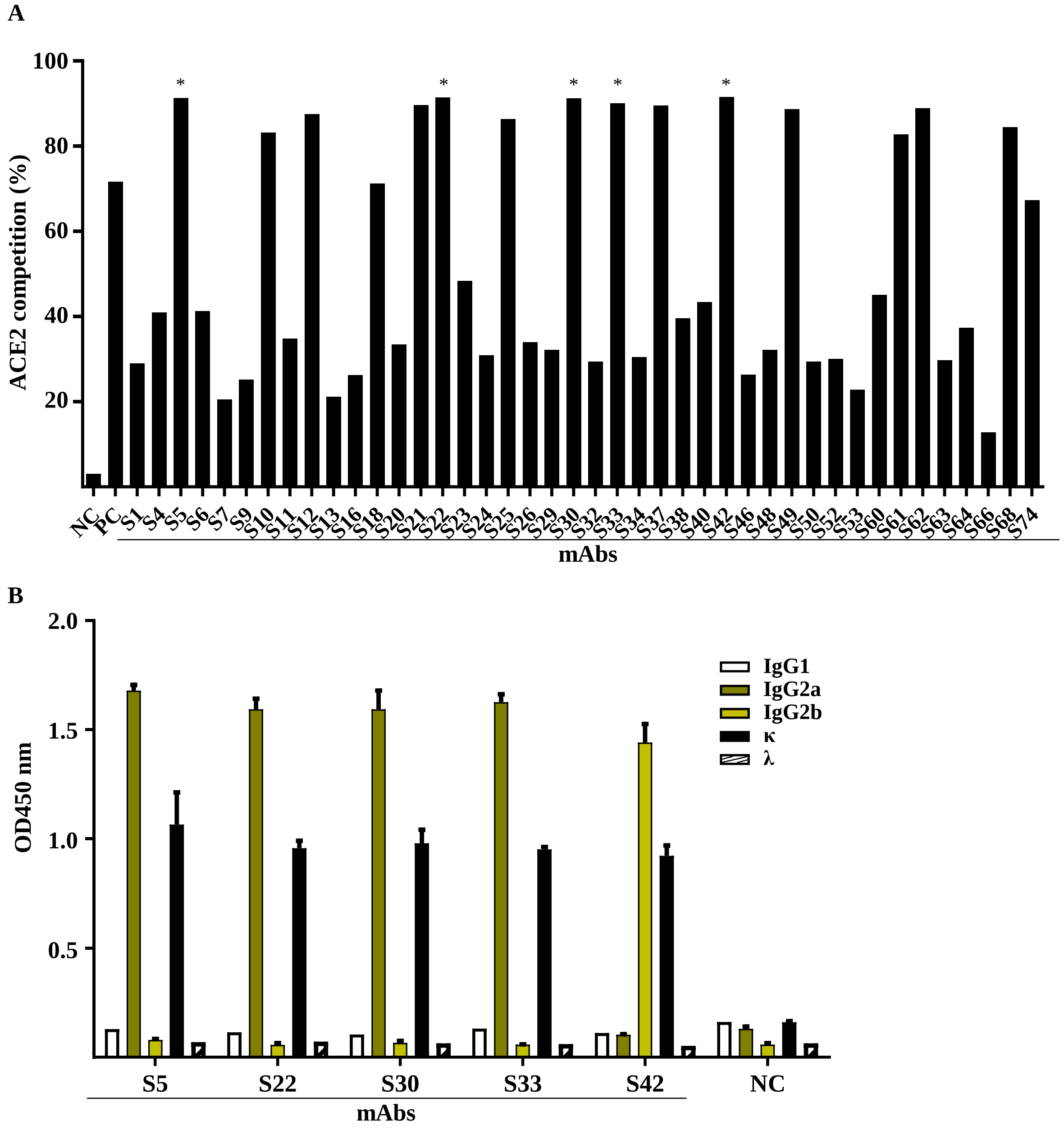

Figure S1. Selection and characterization of monoclonal antibodies against SARS-CoV-2 spike protein. (A) Selected hybridoma supernatants were mixed with 5 nM of biotinylated ACE2 receptor protein and competed with S protein. Purified mouse IgG was used as a negative and positive control, respectively. *: >90% inhibition. (B) Isotypes of mAbs were determined by ELISA. mAbs (1 μg/ml) were analyzed by HRP conjugated goat anti-mouse subclasses, IgG1 (blank), 2a (black), 2b (gray), and light chains κ (light gray) and λ (slash). The results are shown as the mean ± standard errors of the means from duplicate wells.
