# Supplementary material for: Induction of high affinity monoclonal antibodies against SARS-CoV-2 variant infection using a DNA prime-protein boost strategy
Source: J Biomed Sci. 2022 Jun 9;29:37. doi: 10.1186/s12929-022-00823-0 (PMC9178533; doi:10.1186/s12929-022-00823-0)
Supplement: Supplementary file 2 — Additional file 2: Figure S2. The neutralization potency of 5 mAbs against SARS-CoV-2 and its variants. [file 12929_2022_823_MOESM2_ESM.pdf]

Figure S2

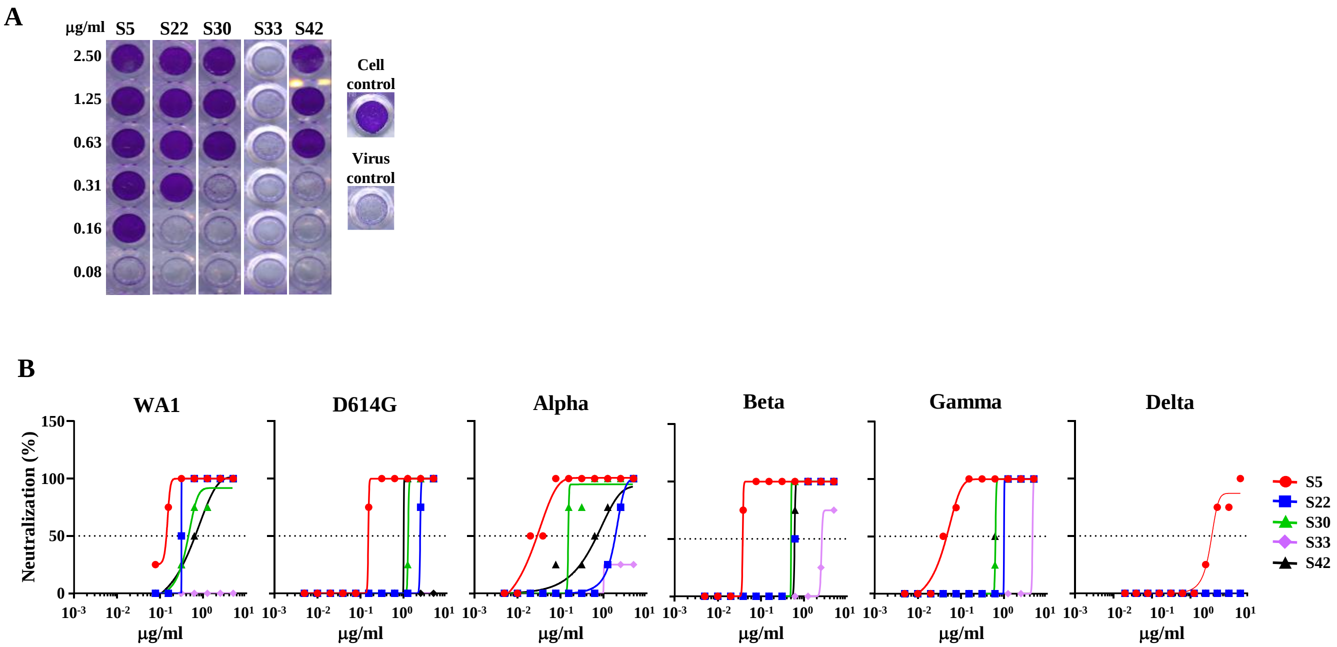

Figure S2. The neutralization potency of 5 mAbs against SARS-CoV-2 and its variants. (A) The viruse (WA1) were mixed with mAbs (a 2-fold serial dilution), incubated for 2 hrs, and subsequently cocultured with Vero cells for 4 days. Representative wells were fixed with 4% formaldehyde and stained with 0.5% crystal violet. (B) Quadruplicates of each dilution were performed. Cytopathogenic effects of viral infection were visually scored for each well. The results were transformed into the percentage of neutralization at a particular monoclonal antibody concentration.
