# Supplementary material for: Induction of high affinity monoclonal antibodies against SARS-CoV-2 variant infection using a DNA prime-protein boost strategy
Source: J Biomed Sci. 2022 Jun 9;29:37. doi: 10.1186/s12929-022-00823-0 (PMC9178533; doi:10.1186/s12929-022-00823-0)
Supplement: Supplementary file 3 — Additional file 3: Figure S3. Competition of human ACE2 protein with mAbs targeting the S proteins of SARS-CoV-2 variants. [file 12929_2022_823_MOESM3_ESM.pdf]

Figure S3

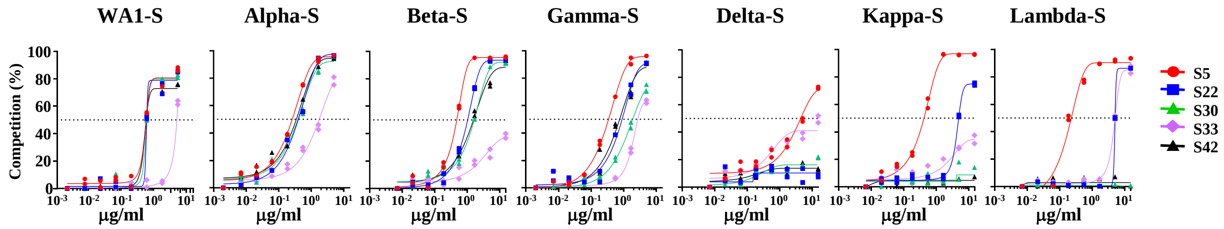

Figure S3. Competition of human ACE2 protein with mAbs targeting the S proteins of SARS-CoV-2 variants. Comparisons of the biotinylated human ACE2 protein and mAbs binding to S proteins of SARS-CoV-2 variants measured using ELISA. The dotted line indicates 50% competition with ACE2.
