# Supplementary material for: Induction of high affinity monoclonal antibodies against SARS-CoV-2 variant infection using a DNA prime-protein boost strategy
Source: J Biomed Sci. 2022 Jun 9;29:37. doi: 10.1186/s12929-022-00823-0 (PMC9178533; doi:10.1186/s12929-022-00823-0)
Supplement: Supplementary file 4 — Additional file 4: Figure S4. Characterization of mAbs against recombinant SARS-CoV-1 RBD306-527. [file 12929_2022_823_MOESM4_ESM.pdf]

Figure S4

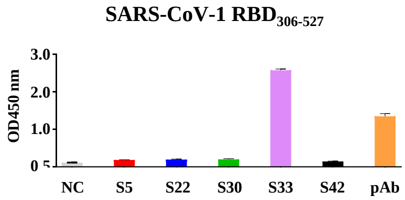

Figure S4. Characterization of mAbs against recombinant SARS-CoV-1 RBD$_{306\text{-}527}$. Reactivity of mAbs (1 μg/mL) toward SARS-CoV-1 RBD$_{306\text{-}527}$ was evaluated using an ELISA reader. The results are presented as the means ± standard errors of the means from triplicate wells..
