# Supplementary material for: Induction of high affinity monoclonal antibodies against SARS-CoV-2 variant infection using a DNA prime-protein boost strategy
Source: J Biomed Sci. 2022 Jun 9;29:37. doi: 10.1186/s12929-022-00823-0 (PMC9178533; doi:10.1186/s12929-022-00823-0)
Supplement: Supplementary file 5 — Additional file 5: Figure S5. Characterization of mAbs against human other coronaviruses. [file 12929_2022_823_MOESM5_ESM.pdf]

Figure S5

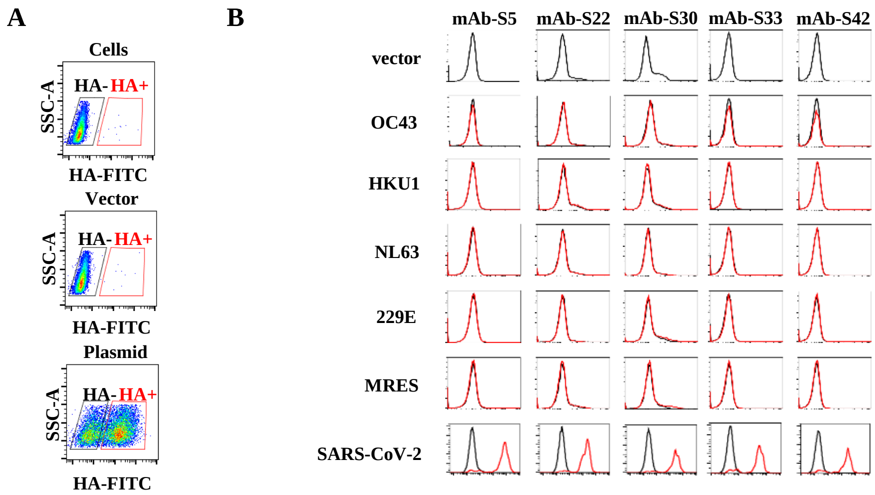

Figure S5. Characterization of mAbs against human other coronaviruses. The full-length of human coronaviral spike genes were optimized for mammalian codon usage and synthesized by GenScript Biotech. All spike genes with HA-tag sequence were subcloned into the expression vectors and transfected into 293T cells for 48 hours. The cells were harvested and stained with FITC-labeled anti-HA and Alexa647-labeled mAbs. The spike gene of SARS-CoV-2 (accession number: DQ412574), MERS-CoV (accession number: KJ782549.1), human OC43 (accession number: KF572815.1), HKU1 (accession number: DQ437607.1), 229E (accession number: AB691763.1), and NL63 (accession number: KM055633.1) were used. (A) Representative gating strategy for transfected 293T cells. (B) Recognition of human coronaviral spike protein by mAbs was determined using flow cytometry. HA^- cells: black line; HA^+ cells : red line.
