# Supplementary material for: Induction of high affinity monoclonal antibodies against SARS-CoV-2 variant infection using a DNA prime-protein boost strategy
Source: J Biomed Sci. 2022 Jun 9;29:37. doi: 10.1186/s12929-022-00823-0 (PMC9178533; doi:10.1186/s12929-022-00823-0)
Supplement: Supplementary file 6 — Additional file 6: Figure S6. The neutralization potency of 5 mAbs against Omicron pseudovirus. [file 12929_2022_823_MOESM6_ESM.pdf]

Figure S6

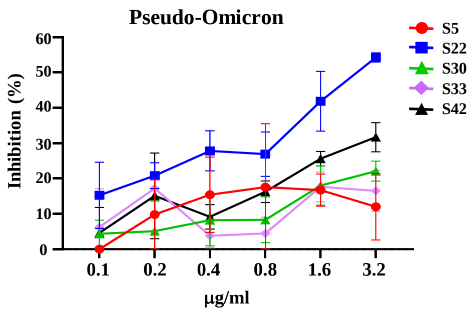

Figure S6. The neutralization potency of 5 mAbs against Omicron pseudovirus. The mAbs mixed with $S^{Omicron}$EM-LvFLuc viruses (4x10^{8} vRNA copies/assay) for 1 h at 37°C. The mixture was added to the BHK21/hACE2 cells in triplicate and incubated at 37°C for another 6 h. The relative infectivity was measured by the Luciferase Assay System (E1501, Promega) with the GLOMAX Multi+ Microplate Multimode Reader (Promega) at 48 hrs. p.i. and calculated.
